# Prevalence of Neuropathic Pain and Related Characteristics in Hidradenitis Suppurativa: A Cross-Sectional Study

**DOI:** 10.3390/jcm9124046

**Published:** 2020-12-15

**Authors:** Simone Garcovich, Simona Muratori, Chiara Moltrasio, Agata Alba Buscemi, Giulia Giovanardi, Dalma Malvaso, Enrico Di Stasio, Angelo Valerio Marzano, Ketty Peris

**Affiliations:** 1 Dermatologia, Fondazione Policlinico Universitario A. Gemelli IRCCS, 00168 Rome, Italy; giu.giovanardi@gmail.com (G.G.); malvasodalma@gmail.com (D.M.); ketty.peris@unicatt.it (K.P.); 2 Dermatologia, Università Cattolica del Sacro Cuore, 00168 Rome, Italy; 3 Dermatology Unit, Fondazione IRCCS Ca’ Granda Ospedale Maggiore Policlinico, 20122 Milan, Italy; simona.muratori@policlinico.mi.it (S.M.); chiara.moltrasio@policlinico.mi.it (C.M.); angelo.marzano@unimi.it (A.V.M.); 4 Department of Medical Surgical and Health Sciences, University of Trieste, 34127 Trieste, Italy; 5 Department of Physiopathology and Transplantation, Università degli Studi di Milano, 20122 Milan, Italy; agataalbamariadomenica@gmail.com; 6 Dipartimento di Scienze Biotecnologiche di Base, Cliniche Intensivologiche e Perioperatorie, Università Cattolica del Sacro Cuore, 00168 Roma, Italy; enrico.distasio@unicatt.it; 7 Dipartimento di Scienze Laboratoristiche ed Infettivologiche, Fondazione Policlinico Universitario A. Gemelli IRCCS, 00168 Roma, Italy

**Keywords:** hidradenitis suppurativa, chronic pain, neuropathic pain, pain management, pruritus

## Abstract

Background: Pain is a core symptom of hidradenitis suppurativa (HS) and is of complex, multifactorial origin. HS patients frequently report typical neuropathic pain qualities, but its prevalence has been poorly described. Methods: In this cross-sectional study, we examine the prevalence of neuropathic pain (NP) component and related pain-characteristics of a hospital-based cohort of patients with symptomatic HS. We administered the pain-DETECT tool (PDQ), a validated screening tool for NP, collecting clinical and patient-reported data on pain, pruritus and pain-management. We obtained 110 complete datasets from symptomatic HS patients (49.1% females; Hurley I (27.3%])–II (45.5%)–III (27.3%)). According to the PDQ tool, 30% of patients were classified with a high probability (>90%) of neuropathic pain (LNP). LNP status was significantly associated with increased pain severity, disease activity, pruritus intensity and use of pain medication. Regression analysis showed a significant impact of the PDQ score on patient-reported outcomes, including pain severity and the dimensions of activity and affective pain interference. HS patients may present a mixed chronic pain phenotype with a neuropathic component, thus requiring additional pain-assessments. A multi-modal approach to pain management, in combination with disease-specific treatment, should be implemented in future interventional studies.

## 1. Introduction

Hidradenitis suppurativa (HS) is a chronic, auto-inflammatory skin disease characterized by painful, deep seated, highly inflamed nodules and draining tunnels in the intertriginous areas of the body [1,2,3]. Pain is a core symptom of HS and is of complex, multifactorial origin [4,5]. Neuropathic pain is defined as pain initiated or caused by a primary lesion or dysfunction in the central and/or peripheral nervous system” and can be induced in chronic pain states by inflammatory and neuroplastic mechanisms [6]. HS patients frequently report typical neuropathic pain-qualities, such as “shooting”, “itchy”, “blinding”, “stinging” and “burning” sensations, as well as chronic pruritus [7]. The prevalence of a neuropathic pain component in the HS patient population is poorly described but has been consistently reported in other chronic inflammatory skin conditions, such as psoriasis, psoriatic arthritis and atopic dermatitis [8,9,10]. Despite the central role of pain as a relevant outcome measure in clinical trials, therapeutic management of pain in HS patients is limited and current clinical practice guidelines (CPGs) do offer only generic advice on specific pain medications [11,12]. Recently, Savage et al. proposed a tailored treatment algorithm for HS-related pain, stratifying interventions on the basis of pain severity and nociceptive/neuropathic pain components. [13].

Among licensed therapies for HS, only adalimumab, a TNF-alpha antagonist, has been shown to reduce HS-related skin pain both in the short and long-term [14]. Patients with HS frequently adopt a variety of pharmacologic and non-pharmacologic/physical or behavioral management strategies to alleviate HS-related skin pain, further highlighting the need for specific treatments [13,15]. The objective of this cross-sectional study was to screen for a neuropathic pain component and evaluate related pain and pruritus outcomes in patients with symptomatic HS during routine clinical care.

## 2. Experimental Section

### 2.1. Methodology

During a six months period, all patients with a diagnosis of HS attending the specialized outpatient clinics of the Dermatology units of Fondazione Policlinico A. Gemelli IRCCS, Rome and Fondazione IRCCS Ca’ Granda, Ospedale Maggiore Policlinico, Milan, were evaluated for this cross-sectional study. Inclusion criteria for cases were the following: male or female adult patients diagnosed with painful HS disease, presenting active, painful clinical lesions and/or at least one inflammatory episode in the last 24 weeks, undergoing regular follow-up and already enrolled in ongoing institutional patient registries for HS.

Patients with long-term stable disease (no flares or pain-free for at least six months) and with a diagnosis of neurologic/psychiatric conditions or in stable treatment with psychoactive medications (antidepressants, anxiolytics) were excluded. Disease-specific data were retrieved from the local patient-registry, including standard demographic information, HS-specific clinical information and outcome measures (disease duration, diagnostic delay, Hurley stage, anatomical distribution of affected body areas), concomitant medication, including pain-specific medication, and comorbidities [16]. HS severity was assessed by a dermatologist during routine physical examination using the Hurley staging classification and International Hidradenitis Suppurativa Severity Score System (IHS4) scoring system, involving the counting of specific inflammatory lesions (nodules, abscesses, fistulas) in affected body regions. The Hurley staging system classifies patients into mild (Hurley I), moderate (Hurley II) and severe (Hurley III) disease, based on the presence of sinus tract/subcutaneous fistulas and the extent of regional involvement.

Number of skin areas involved by HS and symptomatic body regions, with patient-reported pain and/or pruritus, were also recorded and captured during routine clinical activities. The patients completed a series of validated patient-reported clinical outcome measures (PROMs) to collect information on skin-related neuropathic pain and pruritus. These PROMs are already in use during regular patient consultations, capturing baseline symptoms with a seven days-recall period, and include:

- Pain-DETECT questionnaire (PDQ) [10]—The pain-DETECT questionnaire (PDQ) is a symptom-based assessment tool developed to screen for neuropathic pain-like component in chronic mixed-pain states and has been used previously in a variety of conditions, including musculoskeletal disorders, inflammatory joint disease (psoriatic arthritis, rheumatoid arthritis) and chronic cutaneous conditions with skin-related pain and skin-stiffness (psoriasis, pachyonychia congenita, systemic sclerosis). PDQ has both a sensitivity and specificity of 84% and is routinely used in clinical care to screen patients for neuropathic pain [17]. According to the PDQ-score, patient-reported pain is classified into three groups: a score < 13 indicates that the presence of a neuropathic pain component is unlikely (“unlikely neuropathic” or UNP), a score of 13–18 that it is indecisive (classified as “possible neuropathic” or PNP), while a score > 18 indicates that presence of a neuropathic pain component is likely (“likely neuropathic” or LNP) [18].
- Brief Pain inventory short form (BPI) is a self-administered questionnaire designed to evaluate pain severity and its functional consequences. Items of the BPI are scored on a numerical rating scale (0 to 10), measuring the level of pain intensity and related interference with general activity (i.e., mood, walking ability, normal work, relationships with others, sleep, and enjoyment of life) [19,20,21]. The composite score “pain-severity” was constructed as a mean of the four pain intensity items of the BPI (baseline, worst, weakest, mean). The two dimensions of pain-interference were derived as a composite score (mean) of the “activity” (walking, work, general activity) and “affective” items (mood, relation with others, enjoyment of life, sleep) of the BPI, as described previously [22]. Pain-relief received by medications or treatments during the last week’s treatments was rated on a numerical scale 0 (no relief)–10 (complete relief) point scale [22].
- Numerical rating (NRS) intensity scale for pain and pruritus—Pain and pruritus intensity were evaluated by the patient using a numerical rating scale (NRS), at baseline (at the time of consultation) and with a recall period of seven days for the worst, mean and weakest intensities [23].

Additional information on pain-specific aspects was collected, including pain history/duration, frequency/duration of pain attacks, number of “bed days” (0, 5 or more days with forced bed rest and lost work days, excluding hospitalization) due to HS-related pain in the last 12 weeks, use of pain medication, self-reported non-pharmacologic measures of pain management, referral to a pain specialist, use of conservative-medical treatments for management of HS disease, and use of surgery for HS. Information on pain course (classified as persistent pain with slight fluctuations, persistent pain with pain attacks, pain attacks without pain between them, pain attacks with pain between them) and presence of radiating pain was extracted from the PDQ and reported for descriptive statistics.

Because the study was based on an observational design with collection of routine clinical data in local HS-patient registries only, a notification to the Ethical Committee of the two centers was requested.

### 2.2. Statistical Analysis

Continuous data were reported with median and interquartile range (IQR), and categorical data were reported with a frequency distribution. The data was tested for normality using Shapiro-Wilk. Differences among PDQ groups-responders, for continuous variables, were tested using the parametric and non-parametric Kruskall-Wallis one-way analysis of variance on ranks, followed by a Dunn’s post-hoc test. Differences in distribution of categorical variables were tested with Chi-square test. Spearman coefficient correlation testing was carried out to analyze correlation between the PDQ-score and clinical parameters (disease duration, IHS4 score) as well as between PDQ-score and PROMs of interest (baseline and worst pruritus NRS intensity score, BPI-pain severity, activity, and affective interference). The effect of the PDQ-score was determined using multivariable linear regression models, with the outcomes of the BPI questionnaire as dependent variables: pain severity, activity interference and affective interference. In the multivariable models, the effect of the PDQ-score was adjusted by sex, age, Hurley stage, extent of painful diseased body areas (> three disease body areas involved), use of any HS-specific medication (topical or systemic) and use of biologic treatment (adalimumab). Selection of these co-factors was based on clinical significance and previous evidence from studies on HS-related pain and pruritus. Independent variables were entered in the model with a backwards approach. Multivariable linear regression was used to assess the effect of PDQ score on baseline pruritus intensity score as the dependent variable, adjusting for the cofactors: sex, age, IHS4 score, extent of painful diseased body areas (>3 body areas), use of any HS-specific medication (topical or systemic) and use of biologic treatment (adalimumab).

## 3. Results

### 3.1. Patient Demographic and Clinical Characteristics

During the time period from 1 February 2019 to 30 July 2019, and during the routine clinical care of 380 patients, we enrolled 110 patients with active-symptomatic disease and with complete datasets: 54 (49.1%) females; 30 Hurley I patients (27.3%), 50 Hurley II (45.5%) and 30 Hurley III patients (27.3%). Demographic and clinical characteristics of the enrolled patient cohort are summarized in Table 1. More than two-thirds of patients presented moderate-severe HS disease (according to Hurley staging) (73.8%), widespread disease (62.7%), involving three or more body regions, and long disease duration (mean 9.9 years). Mean IHS4 score was 10.0, consistent with moderate-severe disease. 60% of patients were under active treatment (topical or systemic) for HS disease, while 27.3% of patients received a biological treatment (adalimumab).

### 3.2. Classification According to the PDQ Scoring System

According to the PDQ, 33 (30%) of 110 patients were classified as “likely neuropathic pain (LNP)”, presenting a PDQ score greater than 18. Thirty-two (29.1%) of 110 patients were classified as having a “possible neuropathic pain” (PNP) component (PDQ score range 13–18), while 45 (40.9%) patients, scoring less than 13 in the PDQ, were considered as having “unlikely neuropathic pain” (UNP) (see Table 1). There were no differences in terms of age, family history, smoking status and BMI across PDQ classification groups (see Table 1). A higher number of female patients was observed in the PNP group (38.9%) (*p* = 0.015). Clinical characteristics such extent of disease involvement and disease duration were not different between PDQ groups (Table 1). Differences in the distribution of Hurley stage across the PDQ groups approached the borderline of significance (*p* = 0.066). Disease severity, as measured by the IHS4 score, was significantly different across PDQ groups (*p* = 0.037), with the LNP group presenting an higher disease severity (median IHS4 = 12) than the PNP group (median IHS4 score = 6.5).

### 3.3. Descriptive Characteristics of Skin-Related Pain in Hidradenitis Suppurativa.

More than 80% of patients reported a year-long pain history, in line with the long disease duration of HS and with the chronic pain phenotype. Descriptive features of pain course and pain attacks are summarized in Table 2. Pain course in our patient cohort was mostly represented by pain attacks without pain between them (41.8%), consistent with the chronic-recurrent nature of HS. Almost half (46.3%) of patient had a persistent pain course, with slight fluctuations (22.7%) of pain attacks (23.6%). Radiation of pain, a typical feature of neuropathic pain, was reported by 20% of patients. This information (course pattern, radiating pain) is incorporated in the PDQ scoring system and was not further tested for statistical significance.

In patients experiencing pain attacks, frequency or duration of episodes were not different between PDQ groups. Notably, 73.7% of patients in the LNP group presented pain attacks with duration of 1–3 or more days, possibly indicating disease flares.

### 3.4. Patient-Reported Outcomes on Pain and Pruritus according to PDQ Status

Regarding patient-reported outcomes, LNP responders presented higher baseline and worse pain (*p* < 0.001) in comparison to the PNP- and UNP-groups (see Table 3). For the BPI-assessment measures, patients in the LNP group scored significantly higher in the composite pain severity score (*p* < 0.001) than the PNP- and UNP- groups. Both activity and affective pain interference results were higher in the LNP group (*p* < 0.001) versus the other PDQ groups, pointing at a marked impact on the two dimensions of pain interference. Pruritus was reported by 85 (77.3%) of patients, with no difference between PDQ classes. LNP responders presented higher pruritus intensity scores than PNP and UNP groups (Table 3). Patients in the LNP group presented an increased impact of pain on daily activities, reporting a median of three days/months of functional inactivity (bed days) due to disabling pain (*p* < 0.001). (Table 3).

### 3.5. Correlations between the PDQ Score, Clinical Characteristics and Patient-Reported Outcome Measures

The PDQ score did show a weak correlation with pain severity (r = 0.3777, *p* < 0.0001), activity pain interference (r = 0.331, *p* = 0.004), affective pain interference (r = 0.408, *p* < 0.001), baseline pruritus intensity scores (r = 0.388, *p* < 0.0001), but not with disease activity (IHS4) (*p* = 0.125) and disease duration (*p* = 0.8595) (see Table S1).

### 3.6. Regression Analysis PDQ-Score and Pain-Related PROMs

Multivariate regression analysis revealed PDQ-score to be an independent predictor of the outcomes of the BPI pain severity score (Beta = 0.09, SE = 0.023, *p* = 0.001) (Table S2) and on the functional outcomes, activity interference (Beta = 0.11, SE = 0.034, *p* < 0.001) (Table S3) and affective pain interference (Beta = 0.14, SE = 0.03, *p* < 0.001) (Table S4). Of note, in all three models, the use of biologic treatment was a significant independent predictor of pain severity (Beta = −0.912, SE = 0.36, *p* = 0.012), activity interference (Beta = −1.321, SE = 0.619, *p* = 0.035), and affective interference (Beta = −1.115, SE = 0.557, *p* = 0.047). Hurley stage remained a significant cofactor in the models for the outcomes pain severity (Beta = 0.918, SE = 0.212, *p* < 0.001) and activity interference (Beta = 0.908, SE = 0.323, *p* = 0.005).

### 3.7. Regression Analysis PDQ-Score and Pruritus

On multivariable regression analysis, significant predictors for the patient-reported outcome NRS baseline pruritus included PDQ-score (Beta = 0.129, SE = 0.031, *p* < 0.001) and disease activity (IHS4 score) (Beta = 0.084, SE = 0.026, *p* = 0.001) (as shown in Table S5).

### 3.8. Use of Pain Medication

In our cohort, 34.5% (*n* = 38) of patients reported the use of pain-relief medications and only 10% (*n* = 11) reported a referral to pain specialty clinics. The LNP group was associated with the use of pain-medication (*p* = 0.013) (Table 4). Among pain-medications, non-steroidal anti-inflammatory drugs (acetaminophen-derivatives, COX-1 and COX-2 inhibitors) were the most frequently used (*n* = 23), followed by combination of COX-inhibitors and weak opioid preparations (*n* = 10) and topical anesthetics (*n* = 5). Use of strong opioids (morphine, tapentadol) for HS-related pain was reported only by two patients. Sixteen patients reported the use of adjunctive pain-medication, including low-dose systemic steroids (*n* = 14) and anti-convulsivants (pregabalin) (*n* = 2). Use of specific systemic antipruritic drugs (antihistamines) was not reported. Of note, among self-reported pain management strategies for HS-pain, 24.5% of patients employed cold-based compresses or applications of icepacks. Patients in the LNP group reported less pain-relief from any type of intervention (pain-specific or HS-specific therapeutics) than UNP group (*p* = 0.0254).

## 4. Discussion

The results of this study point at a substantial prevalence of a neuropathic-like pain component in patients with active HS disease. Neuropathic pain is initiated or caused by a primary lesion or dysfunction in the central and/or peripheral nervous system and can be induced in chronic pain states by inflammatory and neuroplastic mechanisms [6]. HS is a complex disease of the hair-follicle bearing inverse skin areas characterized by chronic-recurrent inflammation, and, differently from other common skin diseases (psoriasis, atopic dermatitis), by cutaneous-subcutaneous tissue destruction and fibrotic remodeling [24]. Incidence of neuropathic pain has been reported also in other dermatological conditions, including psoriatic arthritis, atopic dermatitis and pachyonychia congenita [9,25]. In routine care, clinometric tools like the PainDetect questionnaire (PDQ) may help the clinician to screen patients for a neuropathic pain component in a variety of clinical conditions and mixed chronic pain states [26].

The prevalence of a neuropathic pain component in almost one-third of HS patients in our study is in line with the results of a previous retrospective study, also using the PDQ tool. In the study of Huilaja et al. the presence of a “likely” neuropathic pain status was reported in 31.5% of HS patients, especially in cases with psychiatric comorbidities, higher QoL impairment and with moderate-severe pain ratings [8]. In the same study, the use of pain-medication was trending lower in PDQ-negative patients, but there was no information on concomitant HS-specific treatment.

In our study, the presence of a “likely” neuropathic pain (LNP) component presented a significant association with validated patient-reported outcome measures of pain and pruritus. Patients classified as LNP-PDQ reported increased pain and pruritus scores as well as a pronounced interference in daily activities and affective dimension due to chronic pain. Functional impairment, measured as forced bed-rest days, was also markedly increased in patients with LNP status. Subgrouping HS patients on the basis of the PDQ and thus on their individual pain phenotype helped identify cases with a higher burden of pain and pruritus.

The importance of skin-related pain as one of the main outcome measures in HS has been reported only in few observational studies and interventional studies [5,27]. Skin-related HS pain has been mostly considered of an inflammatory-nociceptive nature, due to the localized, chronic-recurrent inflammatory nature of the disease and the release of inflammatory mediators in the inflamed skin [11,28]. A subset of patients with chronic-recurrent and/or more extensive HS disease also report a heterogeneous spectrum of pain-perceptions, with “neuropathic”-like pain qualities (“shooting”, “itchy, “burning” etc.) in 32–39% of cases [7]. Other studies have described the prevalence and characteristics of pruritus in HS patients, with conflicting results [29]. Prevalence of pruritus in symptomatic HS patients has been estimated from 41.7% to 77.5% of patients, with higher intensity scores being associated with severe Hurley 3 disease, extensive disease and pain [30]. In most reports, pain and pruritus intensity scores correlate positively with each other and both symptoms are perceived spatially in the same disease body region in 60–74.9% of patient [31,32]. On histology, eosinophilic and perineural inflammation as well as abnormal mast cell innervation has been shown in both peri-lesional and lesion skin of HS patients, pointing at a potential anatomical correlate of “neuropathic” pain [33]. Lipoxygenase-derived inflammatory mediators, eicosanoids and leukotriene B4 are enriched in HS lesional skin and are known inducers of peripheral inflammation and hyperalgesia [34,35]. During the natural progression of HS disease, more severe lesions like complex fistulas, deep-seated inflammatory plaques and cribriform scars are characterized by extensive, local tissue destruction and fibrotic remodeling, which may both anatomically as well as functionally disrupt the local dermal-epidermal neural networks [36].

The concept of a complex pain phenotype in HS patients, which encompasses a “neuropathic” pain component other than inflammatory-nociceptive, may fit well with the progressive, complex pathogenesis of this disease. Furthermore, in chronic pain states, pain and pruritus can be perceived in parallel or can interact with each other, as both sensations are conveyed by the excitation of cutaneous C-nerve fibers [37]. In our study, pruritus was reported by 77.3% of patients. PDQ-LNP status was significantly associated with pruritus and increased intensity scores, further confirming the potential presence of a neuropathic-like pain component in HS patients. The concept of abnormal pain processing and central sensitization to pain has been also proposed in HS due to the high inflammatory burden and chronicity of the disease [38].

A better understanding of the pathophysiology of pain and pruritus in HS could also improve clinical management, as the use of adjunctive pain medication is poorly described in current CPGs [39]. Differently from previous reports, pain-medication use was fairly limited in our patient cohort, as was the referral to a pain-specialist, highlighting an unmet clinical need in the HS population [27]. Country-specific attitudes and practices toward pain-medication in HS patients could explain these differences, especially regarding the prescription of strong opioids [40]. Among systemic agents, only adalimumab has been shown to consistently control skin-related pain in HS patients [14]. Interestingly, the use of anti-TNF-alpha treatment, including adalimumab, has been previously proposed for the treatment of severe acute sciatica, a typical neuropathic pain condition [41]. Also, the use of adjunct medication to control neuropathic pain and/or chronic pruritus, such as gabapentinoids, antihistamines or systemic steroids, has not been evaluated in clinical studies [13]. Population-level studies have also reported a significant risk of opioid over-prescription and use of recreational drugs in HS patients, underlining the need for adequate and disease-specific management of chronic pain and secondary conditions [42]. Self-reported pain management has been previously described in HS patients and includes both pharmacological and physical/non-pharmacological treatment approaches, reflecting the complex pain phenotype of this condition [15]. Interestingly, the different use of warm- or cold-compresses in our study and in a recent web-based patient survey may reflect the different somatosensory perceptions experienced by patients during the fluctuating disease-course (inflammatory flare vs. chronic state) [12].

There are several limitations to our study design, including the enrolment of a hospital-based cohort of patients, with selection of more severe cases of HS. Furthermore, we did not explore the role of chronic fatigue, psychiatric comorbidity and diminished quality of life in our patient cohort, as these factors could contribute to the disease burden and modulate pain/itch perceptions in these patients. We excluded patients with comorbid neurological/psychiatric conditions and related medications, in order to minimize the role of concomitant medications and other chronic pain states. On the other hand, psychiatric comorbidity, especially mood, bipolar disorder and schizophrenia, has been increasingly linked to HS in population-level studies and may negatively impact on pain and itch perceptions [43,44] Excluding these patients may limit the generalizability of our study findings. Some comorbid conditions, such as type 2 diabetes and arthritis/fibromyalgia, could have been related to neuropathic pain states, but these were present only in a minority of patients in our study. The PDQ may represent an additional tool for subtyping HS patients, as it has been previously used in interventional studies for chronic pain [18]. However, the PDQ needs additional, interdisciplinary validation studies in the HS patient population.

## 5. Conclusions

HS patients may present a complex pain phenotype with a neuropathic pain component, thus requiring additional pain-assessments. HS patients should be screened for skin-related neuropathic pain with a clinometric tool, and eventually undergo additional clinical and neurophysiological assessments to confirm the presence of neuropathic pain. Subgrouping of patients on their basis of their pain phenotypes might improve the management of HS disease, identifying patients in need of adjunct pain medication other than HS-specific therapeutics. Longitudinal follow-up studies in HS patients should also explore the stability of pain-phenotypes and evaluate the role of peripheral or central sensitization to pain and pruritus. A multi-modal approach to pain and pruritus management, in combination with disease-specific treatment, should be implemented in future interventional studies for HS.

## Figures and Tables

**Table 1:** Demographics and clinical characteristics of the hidradenitis suppurativa (HS) patient cohort according to PainDetect status: Unlikely Neuropathic Pain (UNP); Possible Neuropathic pain (PNP); Likely Neuropathic Pain (LNP).

Median (IQR)	All *n* = 110	UNP *n* = 45	PNP *n* = 32	LNP *n* = 33	*p*
Age	30.0 (23.0)	29.0 (28.5)	26.0 (11.5)	34.0 (19.5)	0.371
Female, *n* (%)	54 (49.1)	15 (27.8)	21 (38.9)	18 (33.3)	0.015
Family history of HS, *n* (%)	29 (26.6)	10 (22.2)	10 (31.2)	9 (28.1)	0.659
Current smokers *n* (%)	73 (66.4)	29 (64.4)	19 (59.4)	25 (75.8)	0.353
Body Mass-index	24.69 (5.96)	24.84 (4.67)	24.05 (6.28)	26.16 (7.81)	0.769
Disease duration (years)	8.0 (12.0)	7.0 (12.5)	6.5 (8.5)	10.0 (12.5)	0.394
Hurley stage 1 *n* (%)	30 (27.3)	12 (26.7)	14 (43.7)	4 (12.1)	0.066
Hurley stage 2 *n* (%)	50 (45.5)	19 (42.2)	12 (37.5)	19 (57.6)	
Hurley stage 3 *n* (%)	30 (27.3)	14 (31.1)	6 (18.8)	10 (30.3)	
IHS4 baseline	10.0 (11.0)	8.0 (11.0)	6.5 (8.5)	10.0 (12.5) *	0.037
Nr. of affected body-sites 1-2, *n* (%)	41 (37.3)	17 (37.8)	14 (43.7)	10 (30.3)	0.531
Nr. of affected body-sites 3+, *n* (%)	69 (62.7)	28 (62.2)	18 (56.2)	23 (69.7)	
Nr. of painful body-sites, 1-2, *n* (%)	53 (48.2)	19 (42.2)	19 (59.4)	15 (45.5)	0.309
Nr. of painful body-sites, 3+, *n* (%)	57 (51.8)	26 (57.8)	13 (40.6)	18 (54.5)	
Comorbidities, > 2 conditions, *n* (%)	12 (10.9)	5 (11.1)	5 (15.6)	2 (6.1)	-
Diabetes, *n* (%)	6 (5.5)	3 (6.7)	2 (6.3)	1 (3.0)	-
Inflammatory bowel disease (IBD), *n* (%)	6 (5.5.)	3 (6.7)	2 (6.3)	1 (3.0)	-
Musculo-skeletal disorders, *n* (%)	3 (2.7)	0	3 (9.4)	0	-
HS-specific treatment (any), *n* (%)	66 (60.0)	30 (66.7)	17 (53.1)	19 (57.6)	0.375
Topicals, *n* (%)	5 (4.5)	0	3 (9.4)	2 (6.1)	
Antibiotics/other systemic, *n* (%)	31 (28.3)	13 (28.9)	6 (18.8)	12 (36.4)	0.249
Biologics, *n* (%)	30 (27.3)	16 (35.6)	8 (25.0)	6 (18.2)	0.221

* *p* < 0.05 vs. PNP group. Abbreviations: interquartile range (IQR); International Hidradenitis Suppurativa Severity Score (IHS4).

**Table 2:** Descriptive characteristics of pain in the hidradenitis suppurativa patient cohort according to PainDetect classification status: Unlikely Neuropathic Pain (UNP); Possible Neuropathic pain (PNP); Likely Neuropathic Pain (LNP).

Median (IQR)	All *n* = 110	UNP *n* = 45	PNP *n* = 32	LNP *n* = 33	*p*
Pain history					
0–6 months, *n* (%)	6 (5.1)	2 (4.4)	3 (9.4)	1 (3.0)	0.11
6 months–2 years, *n* (%)	13 (11.8)	10 (22.2)	2 (6.3)	1 (3.0)	
2–5 years, *n* (%)	33 (30.0)	15 (33.3)	9 (28.1)	10 (30.0)	
> 5 years, *n* (%)	58 (52.7)	19 (42.2)	18 (56.3)	21 (63.6)	
Pain course					
Persistent pain with slight fluctuations, *n* (%)	25 (22.7)	13 (28.9)	6 (18.8)	6 (18.2)	
Persistent pain with pain attacks, *n* (%)	26 (23.6)	9 (20.0)	9 (28.1)	8 (24.2)	
Pain attacks without pain between them, *n* (%)	46 (41.8)	22 (48.9)	13 (40.6)	11 (33.3)	
Pain attacks with pain between them, *n* (%)	13 (11.8)	1 (2.2)	4 (12.5)	8 (24.2)	
Radiating pain, *n* (%)	22 (20.0)	6 (13.3)	3 (9.4)	13 (39.4)	
Pain attacks frequency *, *n* (%)					
daily	20 (33.9)	6 (18.2)	8 (44.4)	6 (31.9)	0.231
weekly	12 (20.3)	3 (9.1)	5 (27.8)	4 (21.1)	
monthly	27 (45.7)	14 (42.4)	4 (22.2)	9 (47.4)	
Pain attacks duration *, *n* (%)					
seconds to minutes	16 (27.1)	8 (34.8)	6 (35.3)	2 (10.5)	0.480
hours	8 (13.6)	3 (13.0)	2 (11.8)	3 (15.8)	
1–3 days	19 (32.2)	8 (34.8)	5 (29.4)	6 (31.6)	
> 3 days	16 (27.1)	4 (17.4)	4 (23.5)	8 (42.1)	

Abbreviations: interquartile range (IQR); International Hidradenitis Suppurativa Severity Score (IHS4); * related to the subgroups with pain attacks with or without pain between them.

**Table 3:** Patient-reported outcome measures for pain and pruritus according to the PainDetect classification status: Unlikely Neuropathic Pain (UNP); Possible Neuropathic pain (PNP); Likely Neuropathic Pain (LNP).

Median (IQR)	All *n* = 110	UNP *n* = 45	PNP *n* = 32	LNP *n* = 33	*p*
PainDetect-score (0–38)	14.0 (11.0)	9.0 (3.0)	15.0 (2.0)	22.0 (6.2)	-
BPI Pain severity (0–10)	4.5 (2.7)	4.0 (3.0)	3.4 (3.0)	5.5 (2.6) *	<0.001
NRS pain baseline (0–10)	5.0 (5.0)	5.0 (5.25)	4.5 (4.5)	7.0 (3.5) *	<0.001
NRS worst pain	6.0 (4.0)	6.0 (6.0)	5.0 (3.0)	8.0 (3.25) *	<0.001
NRS weakest pain(0–10)	2.0 (3.0)	1.0 (3.0)	1.0 (3.0)	3.0 (4.0)	0.054
NRS mean pain (0–10)	5.0 (5.0)	4.0 (5.25)	3.0 (3.5)	6.0 (3.25) *	<0.001
BPI activity interference (0–10)	4.2 (2.4)	3.6 (2.2)	3.4 (2.4)	5.7 (2.1) *	<0.001
BPI affective interference (0–10)	3.2 (2.7)	2.8 (2.7)	3.0 (2.6)	4.0 (2.7) *	<0.001
Pruritus, *n* (%)	85 (77.3)	31 (68.9)	22 (68.7)	32 (97.0)	0.342
NRS baseline pruritus (0–10)	2.0 (4.0)	2.0 (3.0)	0.5 (3.0)	4.0 (4.0) *	<0.001
NRS worst pruritus (0–10)	7.0 (4.0)	6.0 (5.75)	5.5 (4.0)	8.0 (2.0) *	0.018
NRS weakest pruritus (0–10)	2.0 (2.0)	1.0 (1.0)	1.5 (2.0)	2.0 (3.0) *	0.004
NRS mean pruritus (0–10)	4.0 (3.0)	3.0 (4.0)	3.0 (3.0)	5.0 (4.0) *	0.001
Nr. of bed days/last 4 weeks	2.0 (4.0)	1.0 (4.25)	1.0 (2.0)	3.0 (6.0) *	<0.001

* *p* < 0.05 vs. UNP and PNP groups. Abbreviations: interquartile range (IQR); Brief Pain inventory (BPI); numerical rating scale (NRS).

**Table 4:** Pain management and self-reported pain relief in the hidradenitis suppurativa patients according to PainDetect status: Unlikely Neuropathic Pain (UNP); Possible Neuropathic pain (PNP); Likely Neuropathic Pain (LNP).

Median (IQR)	All *n* = 110	UNP *n* = 45	PNP *n* = 32	LNP *n* = 33	*p*
Patients on pain medication, *n* (%)	38 (34.5)	12 (26.7)	9 (28.1)	17 (51.5)	0.013
Topical anesthetics, *n*	5	2	-	1	.
NSAIDs, *n*	23	7	6	10	.
Weak opioids ±NSAIDs, *n*	10	3	2	5	.
Strong opioids, *n*	2	0	1	1	.
Systemic steroids *, *n*	14	6	3	5	.
Anti-convulsivants *, *n*	2	0	1	1	.
Pain-specialist referral, *n* (%)	11 (10.0)	4 (8.9)	0	7 (21.2)	.
Pain relief (0–10)	4.5 (4.0)	5.0 (4.2)	4.0 (4.0)	3.0 (5.0)	0.025

Abbreviations: interquartile range (IQR); Nonsteroidal anti-inflammatory drugs (NSAIDs); * adjunctive pain medications.

## Supplementary Materials

The following are available online at https://www.mdpi.com/2077-0383/9/12/4046/s1, Table S1. Spearman’s rank correlation between PainDETECT (PDQ) score with clinical parameters and patient-reported outcome measures.; Table S2. Multivariable linear regression of factors associated with pain severity; Table S3. Multivariable linear regression of factors associated with activity pain interference; Table S4. Multivariable linear regression of factors associated with affective pain interference; Table S5. Multivariable linear regression of factors associated with baseline pruritus intensity.
