# Supplementary material for: Prevalence of Neuropathic Pain and Related Characteristics in Hidradenitis Suppurativa: A Cross-Sectional Study
Source: J Clin Med. 2020 Dec 15;9(12):4046. doi: 10.3390/jcm9124046 (PMC7765202; doi:10.3390/jcm9124046)
Supplement: Supplementary file 1 [file jcm-09-04046-s001.pdf]

Journal of Clinical Medicine

Prevalence of Neuropathic Pain and Related Characteristics in Hidradenitis Suppurativa: A Cross-Sectional Study

Simone Garcovich [1,2,*], Simona Muratori [3], Chiara Moltrasio [3,4], Agata Alba Buscemi [5], Giulia Giovanardi [1,2], Dalma Malvaso [1,2], Enrico Di Stasio [6,7], Angelo Valerio Marzano [3,5,†] and Ketty Peris [1,2,†]

Table S1. Spearman's rank correlation between PainDETECT (PDQ) score with clinical parameters and patient-reported outcome measures.

Assessment parameter	Correlation coefficients	*p*
IHS4 score	0.122	0.205
disease duration	0.049	0.609
BPI-pain severity	0.377	< 0.001
BPI-activity pain interference	0.333	< 0.001
BPI-affective pain interference	0.401	< 0.001
NRS pruritus, baseline	0.335	0.001
NRS pruritus, worst	0.196	0.072

Abbreviations: interquartile range (IQR); Brief Pain inventory (BPI); numerical rating scale (NRS).

Table S2. Multivariable linear regression of factors associated with pain severity.

Reduced Model of the regression obtained with a backward method $R^2 = 0.33$			
**Covariate**	**Correlation coefficients**	**Standard Error**	***p***
PDQ score	0.090	0.023	0.001
Hurley stage	0.918	0.212	< 0.001
Use of biologics	−0.912	0.360	0.012

Dependent variable: Brief Pain Inventory (BPI) pain severity; Covariates: PDQ score, sex, age, Hurley stage, disease duration, extent of painful diseased body areas (> 3 body areas), use of any HS-specific medication and biologic treatment (adalimumab). Abbreviations: PainDetect questionnaire (PDQ); Brief Pain inventory (BPI); numerical rating scale (NRS).

Table S3. Multivariable linear regression of factors associated with activity pain interference.

Reduced Model of the regression obtained with a backward method $R^2 = 0.20$			
**Covariate**	**Correlation coefficients**	**Standard Error**	***p***
PDQ score	0.110	0.034	< 0.001
Hurley stage	0.908	0.323	0.005
Use of biologics	−1.321	0.619	0.035

Dependent variable: Brief Pain Inventory (BPI) activity interference Covariates: PDQ score, sex, age, Hurley stage, disease, extent of painful diseased body areas (> 3 body areas), use of any HS-specific medication and biologic treatment (adalimumab). Abbreviations: PainDetect questionnaire (PDQ); Brief Pain inventory (BPI); numerical rating scale (NRS).

Table S4. Multivariable linear regression of factors associated with affective pain interference.

Reduced Model of the regression obtained with a backward method $R^2 = 0.30$			
**Covariate**	**Correlation coefficients**	**Standard Error**	***p***

PDQ score	0.140	0.030	< 0.001
Age	0.039	0.016	0.014
Any HS-specific medication	1.447	0.489	0.004
Use of biologics	−1.115	0.557	0.047

Dependent variable: Brief Pain Inventory (BPI) affective interference Covariates: PDQ score, sex, age, Hurley stage, disease, extent of painful diseased body areas (> 3 body areas), use of any HS-specific medication and biologic treatment (adalimumab). Abbreviations: PainDetect questionnaire (PDQ).

Table S5. Multivariable linear regression of factors associated with baseline pruritus intensity.

**Reduced Model of the regression obtained with a backward method R^2 = 0.22**			
**Covariate**	**Correlation coefficients**	**Standard error**	***p***
PDQ class	0.129	0.031	< 0.001
IHS4 score	0.084	0.026	0.001

Dependent variable: Baseline pruritus intensity score (NRS). Covariates: PDQ score, sex, age, IHS4 score, extent of diseased body areas (> 3 body areas), use of any HS-specific medication (topical or systemic) and use of biologic treatment (adalimumab). Abbreviations: PainDetect questionnaire (PDQ); Numerical rating scale (NRS).
